# Multiwalled Carbon Nanotubes as Nanomaterial Tool in the Management of Prostate Cancer: A Possible Nanoformulation Approach

**DOI:** 10.34172/apb.2022.053

**Published:** 2021-09-29

**Authors:** Raja Murugesan, Raman Sureshkumar, Arun Radhakrishnan, Srikanth Jupudi, Manisha Chennu

**Affiliations:** ^1^Department of Pharmaceutics, JSS College of Pharmacy, JSS Academy of Higher Education & Research, Ooty, India.; ^2^Department of Pharmaceutical Chemistry, JSS College of Pharmacy, JSS Academy of Higher Education & Research, Ooty, India.; ^3^Department of Pharmacology, JSS College of Pharmacy, JSS Academy of Higher Education & Research, Ooty, India.

**Keywords:** Prostate cancer, Drug delivery, In silico studies, Carbon nanotubes

## Abstract

Prostate cancer (PCa) is one of the leading diseases in men all over the world caused due to over-expression of prostate-specific membrane antigen (PSMA). Currently, the detection and targeting of PCa is one of the major challenges in the prostate gland. Therefore, Bruton tyrosine kinase inhibitor molecules like ibrutinib (Ibr) loaded with nanomaterials like multi-walled carbon nanotubes (MWCNTs), which has good physico-chemical properties may be the best regimen to treat PCa. In this strategy, the chemically modified MWCNTs have excellent ‘Biosensing’ properties makes it easy for detecting PCa without fluorescent agent and thus targets particular site of PCa. In the present study, Ibr/MWCNTs conjugated with T_30_ oligonucleotide may selectively target and inhibit PSMA thereby reduce the over-expression in PCa. Hence, the proposed formulation design can extensively reduce the dosage regimen without any toxic effect. Additionally, the present hypothesis also revealed the binding mode of Ibr in the catalytic pocket of PSMA by *in silico* method. Therefore, we presume that if this hypothesis proves correct, it becomes an additional novel tool and one of the conceivable therapeutic options in treating PCa.

## Introduction

Prostate cancer (PCa) is one of the most common cancer type in men and most prevalent in western countries like Europe, the USA, and worldwide.^1-3^ According to ‘GLOBOCON survey-2018,’ the rate of affected people increasing to 1.38 million and the mortality rate rises to 3.8%. The mortality and prevalence level of PCa increasing due to lifestyle changes, environmental factors and many genetic factors. Therefore, PCa has become one of the major life-threatening diseases compared with other cancer diseases. Therefore, PCa is considered to be one of the life-threatening diseases as like other cancer types.^4,5^

The pathophysiology of PCa involves the over-expression of either prostate-specific membrane antigen (PSMA; Type II membrane protein) or glutamate carboxypeptidase II (GCPII). The expression of PSMA increases due to the metabolism of androgen receptor (AR) and modification of insulin-like growth factor-1. Moreover, the primary risk factor of PCa is associated with increased levels of sex hormone-like testosterone (T), dihydrotestosterone and decreased levels of plasma level in sex hormone binding globulin,^6-9^ the cause behind these are believed to be life style changes, food habits (obesity) and genetic factors, which encompasses mechanism variations due to life style changes, food habits (obesity) and genetic factors.^10-16^

Recently, Bruton’s tyrosine kinase (BTK) inhibitors have been reported to play a major role in the radiosensitization of PCa.^17^ Similarly, B-cell receptor (BCR) pathways were also reported to be involved in the implication of multiple signal transduction pathways, which include modifiable, survival, activation, proliferation, and segregation of B lymphocytes (hematopoietic cells).^18-22^ Ibrutinib (Ibr), a BTK inhibitor, involves inhibition of multiple tyrosine kinase receptors, which is proven by *in vitro* cell line and *in vivo* xenograft studies in various cancer models like LnCaP and DU145. Also, similar studies reveal that human PCa cells and mutations in the BTK gene lead to B cell deﬁciency which further exhibited X-linked gamma globulinemia in humans, X-linked immune deﬁciency in mice from multiple organs as well.^20^ The Ibr comprises good potential as radio-sensitizing effects for ‘BTK-BCR’s signaling pathways.^18,19^ Furthermore, Ibr binds to Cys481 of BTK, thereby inhibits kinase activity in the prostate gland.^23^ Generally, it also inhibits autophosphorylation activity, PLCg2 activation, enzyme inactive conformation and other downstream substrates of BTK in B cells.^24-26^

The main objective of PCa treatment is to endorse the higher concentration of drug in the PSMA. The drawbacks of the prostate gland are linked with tightly packed epithelial cells and thus over-expressed with multi-drug resistance proteins, making it a potent barrier for the entry of chemicals and other biomolecules. Also, over-expression of PSMA leads to PCa and treatment requires high concentration, which may further result in unwanted side effects and patient non-compliance. Hence to overcome the above mentioned problems/drawbacks, biomaterials like multiwalled carbon nanotubes (MWCNTs) loaded with Ibr could be a therapeutic approach for increasing bioavailability with less toxic effect due to their surface area and physico-chemical properties.

### Why MWCNTs for diagnosis and detection of PCa?

MWCNTs are being reported for different clinical approaches owing to their unique properties for detection and diagnosis. MWCNTs involve in most important role for sensing activity-based diagnosis. PSMA detection is very complicated in the Prostate gland. MWCNTs act as the most important biosensor for detecting the PSA biomarker level by using various detection methods like photothermal therapy and photodynamic therapy. The biosensor is one of the most excellent analytical tool for the detection of biomaterial samples concerning properties like functional group, structure and bio composition. Furthermore, the bio-composition is implicated in the process of electrical signal translation from the nano materials.^27-30^ Also, MWCNTs do not require any fluorescent agent for detection process and also MWCNTs are chemically modified with electrode for biomedical imaging.^31-34^ Besides, it also exhibits admirable optical properties to add to their unique property.

Generally, anticancer drug molecules contain various mechanism of action with therapeutic effect is ascribed to the cytotoxicity effect but also influence killing the normal cells. In this context MWCNTs based drug carrier overcomes the drawback of the conventional treatments by altering biodistribution and Pharmacokinetic and pharmacological properties. MWCNTs render good drug-carrying capacity to target site. Moreover, MWCNTs loaded drug molecules enhances the therapeutic effects by avoiding various factors like first metabolism reaction (*in vivo* process*)* and chemical instability.

## Hypothesis

**Figure 1:**
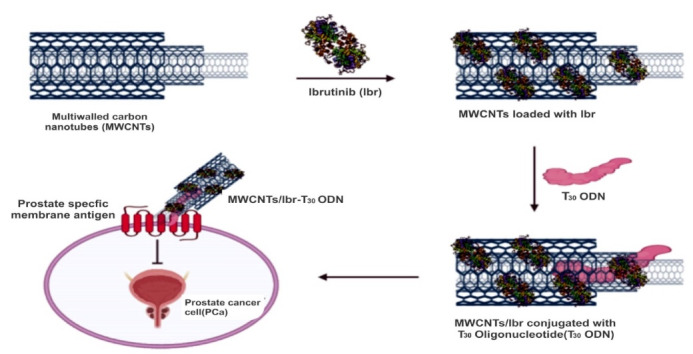
The schematic illustration of Ibr loaded MWCNTs conjugated with T_30_ ODN to target particular site of PSMA for inhibiting PCa cell. The MWCNTs generally have good surface area so Ibr molecules easily loaded with MWCNT by covalent bonding and also it acts as good carrier for targeting particular site and also MWCNTs having Biosensing property to diagnosis. MWCNTs/Ibr conjugation with T_30_ ODN might significantly influence the placing of the drug in the target site of PSMA. Therefore, it appears as a promising solution to penetrate cancer cells and successfully deliver in Ibr.

The present study is to provide an alternative combination of treatments for PCa patients. For this purpose, here we hypothesize MWCNTs loaded with Ibr drug conjugated with T_30_oligonuclotide (T_30_ ODN) used as a carrier to suppress the PSMA, so that it can easily target circulating tumor DNA molecule. Henceforth the drugs incorporated could be effectively delivered to the target site of the prostate. We also presume that targeting the AR could help deliver the drug and suppress it. Furthermore, MWCNTs act as a biosensor and does not require any additional fluorescent agent for bioimaging purpose. In this strategy, MWCNTs-Ibr conjugates with ODN can promote the concentration of test product of interest in the prostate gland and may block the over-expression of PSMA. If our hypothesis works, we propose that PCa’s effective treatment would be a milestone in fighting against prostate-related issues. In this concept, the preliminary *in silico* studies like molecular docking and binding free energy calculation by molecular mechanics generalized born surface area (MM-GBSA), which revealed the binding pose of Ibr in the catalytic pocket of PSMA. The summary of the hypothesis is illustrated in Figure 1.

### The implication of the hypothesis

In this hypothesis, MWCNTs conjugated with oligonucleotide T_30_ (MWCNTs: ODN T_30_) can be used to manage PCa. Though many technologies are used to treat the disease, ultimately, they end up with failure. The present treatment helps in extending the mortality, which depends on the severity of the condition. Among the different cancers affecting the organs, the prostate gland turns to be an untreatable organ due to the negligible amount of the drugs entering the prostate. Hence, using MWCNTs-Ibr conjugated with T_30_ oligonucleotides, might significantly influence the drug’s placing in the target site. Therefore, it appears as a promising solution to penetrate cells and successfully deliver biomolecules. Moreover, the use of MWCNTs as a gene carrier is extensive and is currently growing therefore, the efficiency of the carrier in treating the disease could be enhanced.

## Oligonucleotides inhibit prostate cancer

ODN folds into specific three-dimensional (3D) structures among different dissociation concentrations in the Pico to Nanomolar range.^35^ Moreover, in peculiarity to other nucleic acid molecular probes, aptamers interact and bind to their targets through structural identification. Likewise, ODN recognizes explicitly in a wide range of targets, such as ions, drugs, peptides, toxins, bacteria, viruses, cells, and even tissues,^36-42^ which were mostly concerned in the potential combination therapy of anti-AR in PCa,^43^ The *in vitro* and *in vivo* anticancer activity of several ODN are in pipeline and demonstrated to be potential.^44-48^ Many reports suggest that ODN involves different mechanisms in various organ sites. However, T_30_ ODN may significantly reduce the growth and survival of androgen-independent prostate tumor cells. Henceforth, it also promotes high bioavailability and reduces the toxicity and cell viability for PCa cell lines.^49^

### Justification of Hypothesis –in silico study

**Figure 2:**
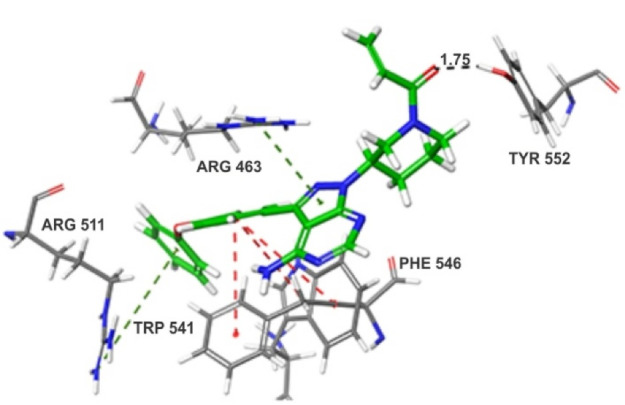
3D binding pose of ibrutinib (Ibr) in the catalytic pocket of PSMA (4LQG.pdb).

The 3D crystal structure of human GCPII in complex with a phosphoramidite inhibitor (4LQG.pdb) was prepared using the protein preparation wizard of Schrodinger 2019-2.^50,51^ Bond orders were refined by the addition of missing hydrogens, loops and sidechains. Protonation and tautomeric states for acidic and basic residues were generated at pH 7.0. Protein minimization was performed using the OPLS3e (optimized potential for liquid simulations) molecular force field^52^ with RMSD of crystallographic heavy atoms kept at 0.30 Å. The structure of Ibr was downloaded from PubChem and LigPrep of Schrödinger suite 2019-2. Low energy conformers were generated, and energy minimized using the OPLS3e force field.^48^ A grid box was generated at the centroid of the co-crystal ligand keeping the van der Waals scaling of 0.8 for the receptor with 0.15 as the partial charge cut-off. Using default parameters of glide,^53^ the LigPrep generated low energy poses were docked into the active site of 4LQG.pdb in extra precision mode (XP). The post docking minimization was performed using prime MM-GBSA (Schrodinger 2019-2), which combines the GBSA continuum solvent model OPLS3e force field model in calculating enthalpy and entropy contribution towards the ligand-protein complex. The glide and MM-GBSA energy results were mentioned in Table 1. From the illustrated Figure 2, the binding pose of Ibr (GScore: -5.60 kcal/mol; Δ_Bind_: -60.36 kcal/mol) formed the majority of hydrophobic interactions with Arg463, Arg511, Trp541, and Phe546. The pyrazole ring of pyrazolopyrimidine ring and phenyl ring formed π-π cation interactions with Arg463 and Arg511, respectively. The phenoxy ring attached at the third position of the pyrazolopyrimidine ring was positioned by forming stable π-π interactions with two hydrophobic residues Trp541 and Phe546. One hydrogen bond was observed between the carbonyl oxygen of the Ibr and the side chain of Tyr552.

**Table 1:** Glide and MMGB-SA energy values (kcal/mol) of Ibr in the catalytic pocket of 4LQG.pdb

**Compound**	**GScore**	**Energy**	**Δ**_Coul_	**Δ**_vdW_	**Δ**_Lipo_	**Δ**_Bind_
Ibrutinib	-5.60	-49.03	-41.76	-36.06	-28.02	-66.36

GScore: glide score; Energy: glide energy; Δ _Coul_: Coulomb energy; Δ_vdW_: van der Waals energy; Δ_Lipo_: Hydrophobic energy; Δ_Bind_: Total binding energy.

The preliminary *in silico* studies like molecular docking and binding free energy calculation by MM-GBSA, which revealed the binding pose of Ibr in the catalytic pocket of PSMA.

## Results and Discussion

With the many research banks, works have been carried out in nanotechnology to deliver anticancer drugs via various nanocarrier systems that arrived in many safety issues. Hence it ushers in new challenges with appreciation for safety and ethical aspects. Previous research shows that many new generation drugs and formulations have entered the pharmaceutical market with potent action with higher cost and more toxic effects. Hence, the present proposed treatment methods end up with severe side effects and ineffective in many cases, which could be due to the above reasons. Henceforth, In recent years to curb such problems, novel/nanoparticulate drug delivery system, either coated polymer or metal/nucleotides complex, have shown an increase in circulation time in the body and reduces the plasma protein adhesion, which has confirmed the enhancement of pharmacokinetic parameters. It also promotes prolonged circulationlife^54^ using MWCNTs. Modification in the delivery system, like targeting the particular site, may improve the treatment and biocompatibility efficacy. Earlier reports suggest that the conjugation of MWCNT with antibody possesses the material’s cellular uptake capability by prostate stem cell antigen is overexpressed cancer cells.^55^ In current work, the *in silico* studies revealed insights into the binding mode of Ibr in the catalytic pocket of glutamate carboxypeptidase II, where the total binding energy of Ibr was favored by the contribution of van der Waals (-36.06 kcal/mol), columbic (-41.76 kcal/mol) and lipophilic energies (-28.02 kcal/mol). In this context, we hypothesize the use of nanotechnology as a tool for targeting to the site-specific prostate gland, which would be a milestone in fighting against prostate tumor tissues. In addition, we discussed the earlier and most recent studies and clinical approaches of MWCNTs in PCa, both detection via biosensor based system and diagonsis via. It promises competent treatment in cost valuable way, it showed lowest amount side effects and also capable of decreasing time period. In addition, the new drug molecules remodel by various nanomaterials source. In this strategy, these biomolecules without difficulty interact with cells. Particularly, involve major role in targeting and drug delivery in both *in vitro* and *in vivo* method.

## Acknowledgments

This work financially supported by the JSS Academy Higher Education and Research (JSSAHER), Mysuru. Award No: REG/DIR(R) /JSSURF/29(1)/2020-11. We also acknowledge the authors would like to thank the Department of science and Technology- Fund for improvement of science and technology infrastructure in universities and Higher Educational Institutions (DST-FIST), New Delhi for their infrastructure support to our department.

## Ethical Issues

Not applicable.

## Conflict of Interest

There is no conflict of interest.
